# Editorial: The future of modern medicine: cells, scaffolds, and biofactors

**DOI:** 10.3389/fmed.2024.1434760

**Published:** 2024-06-27

**Authors:** Laila A. Damiati, Daniel Durand-Herrera, Marwa El Soury

**Affiliations:** ^1^Department of Biological Sciences, College of Science, University of Jeddah, Jeddah, Saudi Arabia; ^2^Multidisciplinary Center for Biotechnology Studies, Faculty of Veterinary Medicine, Universidad Michoacana de San Nicolas de Hidalgo, Morelia, Mexico; ^3^Department of Clinical and Biological Sciences, University of Torino, Torino, Italy; ^4^Neuroscience Institute Cavalieri Ottolenghi (NICO), University of Torino, Orbassano, Italy

**Keywords:** modern medicine, cell therapy, regenerative medicine, personalized medicine, bioengineering, tissue engineering

Modern medicine is undergoing a transformation driven by advances in the field of regenerative medicine, where cells, scaffolds, and biofactors are being developed into innovative therapeutic solutions. To address this significant convergence, this Research Topic explores the potential of these elements in shaping the future of personalized medicine and the impact they will have on patient care. The following articles delve into the intersection of neurobiology and tissue engineering, highlighting the critical role of cellular interactions, stem cells, and advanced diagnostic techniques to enhance tissue regeneration and address critical medical conditions. This Research Topic shows the importance of interdisciplinary approaches and innovative strategies to advance personalized medicine.

Neuro-bone tissue engineering is an interdisciplinary field that combines neurobiology and bone tissue engineering to create innovative approaches to repairing injured bone tissue. The skeletal system is highly innervated, with the peripheral nervous system (PNS) playing a key role in transmitting signals and connecting the central nervous system (CNS) to peripheral organs. The PNS also plays a crucial role in tissue regeneration, as it contains different types of cells such as fibroblasts, macrophages, vasculature-associated cells, and Schwann cells. These cells contain various growth factors such as nerve growth factor (NGF), brain-derived neurotrophic factor (BDNF), glial cell-line-derived neurotrophic factor (GDNF), Neurotrophin-3 (NT-3), and basic fibroblast growth factor (bFGF) that promote a regenerative phenotype. The PNS and CNS work in tandem to regulate bone homeostasis and regeneration through neurogenic and bone factors in the presence of different types of scaffolds that support this regeneration. In this regard, the review by Damiati and El Soury explored the interaction between nerves and bone in various regenerative processes and the strategies for addressing solutions to bone injuries. The authors highlighted where the crosstalk between bone and nerve is crucial for maintaining metabolomic and functional homeostasis. Given this interaction, bone scaffolds are enriched with neurotrophins, neurotransmitters, and neuropeptides, showing a promising strategy for repairing bone fractures.

The periosteum, a thin fibrous layer covering the bone surface, plays a significant role in bone physiology during growth, development, and remodeling. It has gained considerable attention as a source of mesenchymal stem cells (MSCs) due to its ability to generate cells with various chondrogenic, osteogenic, and adipogenic abilities. Periosteum-derived cells (PDCs) represent a promising strategy in tissue engineering. The review by Cao et al. provided an overview of the characterization of PDCs and the procedures used for their isolation. The authors also compared PDCs with other common sources of MSCs, highlighting their unique attributes. The review emphasizes the importance of further research to explore the clinical utility of PDCs and to establish their relative benefits and optimal applications in regenerative medicine.

The rising prevalence of lower back pain, particularly due to intervertebral disc degeneration (IVDD), is a leading cause of disability, especially among the elderly. Current treatments mostly focus on managing symptoms and surgical interventions, such as spinal fusion, which alleviate pain but do not address the root cause and can lead to complications. Recent research has shed light on the role of immune regulation and inflammation in IVDD-induced pain. It is suggested that the activation of the neuroimmune system triggers inflammatory responses that exacerbate the condition. Understanding these mechanisms has led to the exploration of immunomodulatory therapies for IVDD. The review by Gao et al. focused on the interaction of the immune system with IVDD, highlighting the interplay between innate and adaptive immunity and how they contribute to inflammation and tissue damage. Immunomodulatory therapies, such as mesenchymal stem cells (MSCs), small molecules, growth factors, scaffolds, and gene therapy, are discussed as potential treatments. The authors concluded by emphasizing the need for further research to optimize immunomodulatory therapies for IVDD, considering factors such as dosage, donor selection, and individual response variability. Early intervention and bridging the gap between preclinical research and clinical trials are critical to advancing these therapies and improving patient outcomes.

Wound healing is a complex biological process that involves tissue repair after injury. It relies on the bioavailability of growth factors and cytokines to ensure proper cellular responses. Transforming growth factor beta (TGF-β) has shown significant effects on wound healing due to its wide-ranging impact on skin repair. A study by Al-Attar et al. showed the role of TGF-β3 in bone cell wound healing, along with other related factors under both hydrodynamic shear stress and static conditions in cultured bone cells. These findings revealed that under static conditions, a beneficial outcome from TGF-β3 treatment was observed after 24 h in culture, while under hydrodynamic shear stress conditions, TGF-β3 proved to be effective in promoting wound healing. This study concluded that the synergistic effects of TGF-β3 and hydrodynamic shear stress conditions had a positive impact on accelerating wound healing and improving the rate of wound closure, which suggests that incorporating dynamic conditions along with TGF-β3 could enhance the effectiveness of wound healing in bone cells.

Single-cell sequencing is a novel technique that can provide individual cell expression profiles at the genomic, transcriptomic, or epigenomic level. It is a promising tool for detecting cellular heterogeneity and identifying rare subpopulations. Pancreatic cancer is inevitably diagnosed at the late stages of the disease, which increases the need for more effective diagnostic methods. The review by Zhang et al. explores the integration of single-cell sequencing technology into the early diagnosis and treatment of pancreatic cancer. The review discusses the advantages of using single-cell sequencing to detect novel surface markers for circulating tumor cells as an efficient method for early diagnosis. It also provides insight into several therapeutic strategies. A deeper mechanistic understanding of gemcitabine chemotherapy resistance was analyzed by single-cell sequencing, revealing an upregulation of intracellular calcium signaling-related gene expression. This suggests that this pathway is a crucial modulator for effective chemotherapy treatment. Single-cell sequencing analysis suggests a new potential combination of immune checkpoint inhibitor-targeted immunotherapy and tumor-associated macrophages as a novel strategy for pancreatic cancer treatment. Single-cell sequencing analysis has provided an in-depth understanding of cancer-associated fibroblasts (CAFs) and their role in pancreatic ductal adenocarcinoma, highlighting potential therapeutic strategies targeting CAFs and the Hedgehog signaling pathway to improve treatment outcomes. Metastasis is a significant problem in pancreatic cancer, and single-cell sequencing analysis has identified inhibition of epithelial-to-mesenchymal transition as a target for stabilizing tumor cells and preventing their metastasis. Despite the advantages of single-cell sequencing technology in understanding the cellular heterogeneity of pancreatic cancer, there are still some challenges, such as the limited throughput of the platform and the variability in data reliability and reproducibility, that need to be considered. The authors suggest integrating single-cell sequencing with other ‘omics' data, standardizing protocols, and analysis methods, and improving sequencing platforms. These approaches should overcome any limitations and help to realize the full potential of single-cell sequencing in the clinical management of pancreatic cancer.

In summary, the collection of articles on this Research Topic highlights the importance of adapting cells, scaffolds, and growth factors in innovative new treatments to improve patient outcomes. This will help pave the way for a more personalized, patient-centered healthcare landscape in the future.

## Author contributions

LD: Writing – original draft, Writing – review & editing. DD-H: Writing – original draft, Writing – review & editing. ME: Writing – original draft, Writing – review & editing.

